# Gelatin–Tea Tree Essential Oil Coating Improves the Quality, Flavor, and Micromolecular Metabolites of Squid (*Illex argentinus*) Muscle During Cold Storage

**DOI:** 10.3390/foods14071160

**Published:** 2025-03-27

**Authors:** Huijuan Zhou, Jinlin Li, Chengwei Yu, Mingming Hu, Bizhen Zhong, Zongcai Tu, Bin Peng

**Affiliations:** 1 National R&D Branch Center for Conventional Freshwater Fish Processing, College of Life Sciences, Jiangxi Normal University, Nanchang 330022, China; 15103665763@163.com (H.Z.); lijinlin405@126.com (J.L.); 2006abc-hmm@163.com (M.H.); 18070496212@163.com (B.Z.); 004756@jxnu.edu.cn (Z.T.); 2 School of Health, Jiangxi Normal University, Nanchang 330022, China; yuchengwei@jxnu.edu.cn; 3 State Key Laboratory of Food Science and Resources, Nanchang University, Nanchang 330047, China; 4 Engineering Research Center of Freshwater Fish High-Value Utilization of Jiangxi Province, Jiangxi Normal University, Nanchang 330022, China

**Keywords:** squid, refrigeration, coating film, volatile, microorganism diversity

## Abstract

Squid muscle is delicious and nutritious, but it is highly susceptible to spoilage, severely limiting its market development. This study comprehensively evaluated the effects of gelatin (Gel), essential oil (Ess), and Gel + Ess coating on the quality, flavor, and microorganisms of squid muscle during refrigerated storage (4 °C). The results showed that squid muscle treated with Gel + Ess exhibited higher water-holding capacity and sensory evaluation, but lower pH value, chromaticity, and texture parameters than those treated with sterile water (control) during cold storage. The total volatile basic nitrogen (TVB-N) result demonstrated that the shelf life of squid muscle treated with Gel + Ess coating was extended to 12 days at 4 °C, longer than the control group. The Gel + Ess group had the best inhibitory effect on aldehydes and ketones in refrigerated squid muscle, and it could effectively maintain the flavor quality. In addition, the Gel + Ess coating showed an additive inhibition on the growth of *Cockerella* and *Shiwanella* compared to the individual compounds. The Gel + Ess coating is a novel strategy for improving the quality, flavor, and micromolecular metabolites of squid muscle during cold storage.

## 1. Introduction

Squid (*Illex argentinus*), also known as soft fish, belongs to the cephalopod class of mollusks. Recently, squid has become one of the main raw materials for aquatic product processing in China, with an annual output of 0.31 million metric tons in 2023 (China Fisheries Statistical Yearbook 2024) [1]. However, Wang et al. (2023) reported that squid muscle was prone to spoilage and even posed significant safety hazards due to the high water content, high endogenous enzyme activity, and high content of spoilage microorganisms on the surface [2]. At present, the vast majority of processing methods for squid muscles still use traditional preservation methods, such as refrigeration and freezing preservation. Freezing can maintain the freshness of squid, but it also has adverse effects on its quality. Wang et al. (2024) found that freezing caused problems such as changes in taste and loss of nutrients in squid [3]. Therefore, it is of great practical significance for developing simple, easy-to-operate, environmentally friendly, efficient, and safe preservation technologies to extend the storage period of squid muscle.

Coating preservation technology is an emerging preservation technique widely used in the food industry. The main function of the coating is to evenly cover a layer of polymer liquid film on the surface of food, thereby isolating food and air. Wibowo et al. (2024) provided an overview of coating on controlling respiratory metabolism, reducing water loss, and maintaining the quality of stored food [4]. Among them, gelatin (Gel) coating is an effective way of extending the shelf life of meat products for preservation. As a natural polymer substance, gelatin can be evenly applied to the surface of food due to its excellent film-forming and adhesive properties. It forms a dense film that effectively isolates contact with oxygen, moisture, and microorganisms, thereby slowing down the spoilage and deterioration of meat products [5]. In addition, it can also be consumed with meat products with multiple advantages such as safety and good preservation effects [6]. However, as a coating material, gelatin also faces problems such as lack of biological activity, fragile texture, easy breakage, and poor stability, which seriously hinder its further development in the food industry [7]. Therefore, natural essential oil extracts are usually added to gelatin solutions to broaden their applications in the food industry by altering the activity of gelatin coating [8]. Research has shown that natural essential oil (Ess) extracts typically have strong biological effects, such as strong antioxidant and antibacterial activity. Ess extracts effectively inhibit the growth and reproduction of microorganisms, thereby extending the shelf life of meat products [9]. Among them, tea tree essential oil has broad-spectrum antimicrobial activity and antioxidant properties [10]. Tea tree essential oil is widely regarded as a high-quality natural fungicide and preservative. It has been widely used in various industries such as pharmaceuticals and food [11], and is expected to become a new type of biological preservative. Gel and Ess coatings have been reported in relation to keeping the freshness of muscle in seafood [12,13], but knowledge about the effect of Gel + Ess coatings on the refrigerated squid muscle was rather limited. Given the potential benefits of Gel + Ess coatings, this study explores their effect on the preservation of squid muscle.

Microbial-mediated flavor deterioration constitutes a critical determinant of quality degradation in refrigerated aquatic products. Parlapani et al. [14] employed high-throughput sequencing technologies to systematically characterize the dominance of *Psychrophilic* bacteria genera during the refrigeration of squid, postulating their involvement in off-flavors associated with ethyl esters and sulfides. Furthermore, the formation of biogenic amines was seen as a sign of fish spoilage and flavor deterioration. Biogenic amines are mainly caused by protein degradation, amino acid decarboxylation, and carbonyl compound amination reactions caused by the metabolism of spoilage bacteria [15].

In this study, fresh squid muscle samples were subjected to immersion treatment with individual solutions of Gel, Ess, and their composite formulation (Gel + Ess), followed by controlled refrigeration at 4 °C. The effect of the Gel + Ess solution in adjusting the quality deterioration, flavor, and microorganisms of refrigerated squid muscle was investigated. Specifically, this work revealed the underlying mechanism through which Gel + Ess suppresses flavor deterioration in refrigerated squid muscle via microbial community regulation. It will contribute to the application of Gel + Ess coating to the preservation of prepared squid meat products and provide theoretical data.

## 2. Materials and Methods

### 2.1. Materials

Argentine squids (400–600 g) were purchased from China Aquatic Products Zhoushan Marine Fisheries Corporation in Zhoushan, Zhejiang, China. Tea tree essential oil and fish gelatin were purchased from Shanghai Aladdin Biochemical Technology Co., Ltd., Shanghai, China and Shanghai Ye Yi Biotechnology Co., Ltd., Shanghai, China, respectively. Tween-80 was purchased from Beijing Solaibao Technology Co., Ltd., Beijing, China. Magnesium chloride, boric acid, hydrochloride, sodium chloride, and 95% anhydrous ethanol were analytically pure and purchased from Xilong Science Co., Ltd., Guangdong, Shantou, China.

### 2.2. Sample Pretreatment

The frozen squids were thawed at 4 °C for 12 h, and then they were processed to remove blood, internal organs, head, and tail. The squids were cleaned to eliminate surface impurities. Twenty squids were cut into small pieces approximately 3.0 × 1.0 × 1.5 cm (5 ± 0.5 g), then placed individually into clean and sealable bags.

Fish gelatin (4.0 g) was accurately weighed and dissolved in 100 mL of distilled water using a 500 mL borosilicate glass beaker. The mixture was subsequently subjected to continuous mechanical stirring in a thermostatically controlled water bath maintained at 37 ± 0.5 °C for complete dissolution over a period of 1.0 min [16]. A gelatin coating solution with a mass fraction of 4% was prepared (Gel group). Tea tree essential oil (0.4 g) and Tween-80 (0.4 g) [17] were mixed in a 1:1 ratio by mass, using the distilled water as the benchmark (100 mL), resulting in an essential oil coating solution (Ess group). Specifically, equal volumes (100 mL each) of the two solutions (Gel and Ess solution) were combined and thoroughly mixed to form a homogeneous 200 mL gelatin–essential oil coating solution (Gel + Ess group). Distilled water (100 mL) was processed following the same protocol outlined earlier to serve as the blank group.

The squid samples were randomly allocated into four groups, as follows: the blank group, the Gel group, the Ess group, and the Gel + Ess group. Forty-two squid slice samples in each group were immersed in their respective solutions at room temperature for one min until completely submerged. Subsequently, the samples were removed and allowed to sit at 4 °C for 30 min. Once a solid film had formed on the surface of each squid, the samples were packed into clean Ziplock bags, labeled appropriately, and stored at 4 °C for 0, 2, 4, 6, 8, 10, and 12 days (n = 6).

### 2.3. Determination of Water-Holding Capacity

The determination of the water-holding capacity of the samples was based on the method of Yan et al. (2024) with a few modifications [18]. The squid muscle sample weighing 5.00 g was to be completely wrapped with a double layer of qualitative filter paper and placed into a 50 mL centrifuge tube. Subsequently, the test tube containing the squid muscle sample was balanced and inserted into a high-speed refrigerated centrifuge, where it underwent centrifugation at 4 °C and 4000 r/min for 10 min. Following this, the filter paper on the surface of the squid was removed, dried, and then re-weighed to record its weight. The water-holding capacity was calculated using the following formula:

$$\mathrm{WHC}=\frac{m_{1}}{m_{0}}\times 100\%$$

*m*_0_—the weight of the squid before centrifugation, g; *m*_1_—the weight of the squid after centrifugation, g.

### 2.4. pH Assay

The pH values in the squid samples were measured according to the method of Zheng et al. [19] with some modifications. Firstly, the squid sample was minced using a meat grinder. The minced squid muscle sample of 4.00 g was added into a 50 mL centrifuge tube, and 40 mL distilled water was added to evenly disperse using a homogenizer for one min. Subsequently, a high-speed refrigerated centrifuge was employed at 5000 r/min and 4 °C for 5 min to eliminate precipitation and retain the supernatant. The pH value of the centrifuged supernatant was then measured at room temperature using a digital pH meter after stabilization of readings.

### 2.5. Determination of Total Volatile Basic Nitrogen (TVB-N)

The TVB-N in squid samples was determined as previously described by Wu et al. [20] with a slight modification. A meat grinder was used to mince the squid sample until it was pureed. Then, 4000 × *g* of ground squid muscle was accurately weighed and placed at the bottom of the digestion tube, and 20 mL of distilled water was added, evenly dispersed for 5 min with a vortex mixing device, then left for 30 min and impregnated. Subsequently, 1.0 g magnesium oxide was added, and the instrument was quickly connected for determination. The content of TVB-N in the squid sample was calculated by the following formula:

$$X=\frac{(V_{1}-V_{2})\times c\times 14}{m}\times 100$$

*X*—content of TVB-N, expressed in mg/100 g; *V*_1_—sample titration volume of hydrochloric or sulfuric acid standard titration solution (mL); *V*_2_—reagent blank consumption of hydrochloric or sulfuric acid standard titration solution volume (mL); *c*—concentration of a standard titration solution of hydrochloric or sulfuric acid, mol/L; 14—titrating 1.0 mL hydrochloric acid or sulfuric acid standard titrating solution equivalent to the mass of nitrogen, g/mol; *m*—sample mass (g); and 100—calculation results were converted to mg/100 g conversion factor.

### 2.6. Color Measurement

The color measurement of squid samples was conducted according to the description in Aracati et al. [21] with slight modifications. Before measurement, the portable color difference meter (WSC-2B, Kesheng Instrument Co., Ltd., Hangzhou, China) was turned to zero using the standard whiteboard. Three squid blocks with uniform color and flat surfaces were selected for each group. Three points were randomly selected on the squid surface, and then the selected detection points on the squid surface were closely connected for measurement to ensure no light leakage. The L*, a*, b*, and ΔE* values of each group were recorded. Color difference (ΔE*) was calculated by the following equation:

$$\Delta E*=\sqrt{{\left(\Delta L*\right)}^{2}+{\left(\Delta a*\right)}^{2}+{\left(\Delta b*\right)}^{2}}$$

### 2.7. Texture Determination

The texture characteristics of squid samples were determined according to Zheng et al. [19] with slight modifications. Squid samples were divided into small pieces of 1.5 cm × 1.0 cm × 1.5 cm and stood at room temperature for 30 min to eliminate the effects of low temperature. Each sample underwent two axial compressions with a compression ratio of 50% using the same probe; test probe: P/36R; pre-measurement speed: 1 mm/s; test rate: 1 mm/s; post-measurement speed: 1 mm/s; probe 2 measurements with an interval of 5 s; measurement mode: T P.A. Perform 12 repeated tests on each group of samples, and take the average of the results.

### 2.8. Aroma Profile Analysis

The aroma profile of squid samples was determined based on a report by Liang et al. [22] with minor modifications. The squid sample was minced by a meat grinder, and 5.0 g of the meat sample was crushed in a 15 mL sample bottle and placed in a 55 °C water bath for 30 min. Then, the extraction head was vertically inserted into the sample bottle and adsorbed in the headspace for 30 min. After that, the extraction head was removed and quickly inserted into the inlet and desorbed at 250 °C for 10 min, and GC-MS determination was performed.

Chromatographic parameter: inlet temperature 250 °C; the column was DB-WAX capillary column (30 m × 0.25 μm × 0.25 mm). The carrier gas was He; the flow rate was 1.0 mL/min; the non-diversion mode was adopted; the heating procedure was 40 °C for 3 min, and the temperature was raised to 240 °C at 5 °C/min for 15 min. Mass spectrum parameter: EI ionization source; ionization voltage 70 eV; ion source temperature 230 °C; quadrupole temperature 150 °C; the total running time was 54 min.

After the determination, unknown compounds were searched through the NIST 14 mass spectrometry library, and compounds with matching degrees greater than 800 were selected for identification. The chemical name, CAS number, and retention time (RT) of the compounds were determined. The relative content of each volatile substance was calculated by the ratio of peak area. The relative odor activity values (ROAV) were used to evaluate the contribution of volatile organic compounds to flavor [23].

### 2.9. Microorganism Diversity Test

Microbiological analysis of refrigerated muscle was determined according to the procedures described by Li et al. [24] with slight modifications. After genomic DNA extraction in fish samples, the genomic DNA was detected by 1% agarose gel electrophoresis. The V3-V4 hypervariable regions of the bacterial 16S rRNA gene and spike-ins were amplified with the primers 338F (5′-ACTCCTACGGGAGGCAGCA-3′) and 806R (5′-GGACTACHVGGGTWTCTAAT-3′), then sequenced using Illumina NovaSeq 6000 sequencer (Illumina, San Diego, CA, USA). PCR was performed using TransGen AP221-02 (TransGen Biotech Co., Ltd., Beijing, China): TransStart Fastpfu DNA Polymerase. PCR instrument: ABI GeneAmp&reg; Type 9700. PCR products of the same sample were mixed and detected by 2% agarose gel electrophoresis. The PCR products were recovered using AxyPrepDNA gel recovery kit (AXYGEN) (Axygen Scientific, Union City, CA, USA) and elution by Tris-HCl.

The PCR products were quantified using the QuantiFluor™-ST Blue fluorescence quantification system (Promega) (Promega Corporation, Madison, WI, USA) in reference to the initial quantitative results of electrophoresis and then mixed proportionately according to the sequencing volume requirements of each sample. The Illumina official connector sequence was added to the outer end of the target region by PCR; the gel recovery kit was used to cut glue and recover PCR products. Tris-HCl buffer eluted, 2% agarose electrophoresis detection; sodium hydroxide denatures, producing a single strand of DNA. One end of the DNA fragment was complementary to the primer base and fixed to the chip; the DNA fragment was used as a template, the fixed base sequence on the chip was used as a primer for PCR synthesis, and the target DNA fragment was synthesized on the chip. After denaturation by annealing, the other end of the DNA fragment on the chip was randomly complementary with another nearby primer and was fixed to form a “bridge”; PCR amplification to produce DNA clusters; DNA amplicon linearization into a single strand. The modified DNA polymerase and dNTP with four fluorescent labels were added, and only one base was synthesized per cycle; the surface of the reaction plate was scanned by laser to read the types of nucleotides polymerized in the first reaction of each template sequence; the “fluorophores” and “terminating groups” were chemically cut to restore the 3′ end viscosity and continued to polymerize the second nucleotide; statistical results of fluorescence signals collected in each round to obtain the sequence of template DNA fragments (Greengenes database v.13_8).

### 2.10. Statistical Analysis

All experiments were conducted in triplicate with independent biological replicates, and results are presented as mean ± standard deviation (SD). Statistical analysis was performed using SPSS 16.0 software (SPSS Inc., Chicago, IL, USA) through one-way analysis of variance (ANOVA) followed by Duncan’s multiple range test for post hoc comparisons. Significant differences between groups were determined at *p* < 0.05. Microflora analysis was performed by Meiji Bio Yunping (https://cloud.majorbio.com) (accessed on 1 June 2024).

## 3. Results and Discussions

### 3.1. Changes in the Quality of Refrigerated Squid Muscle

#### 3.1.1. Water-Holding Capacity

**Figure 1:**
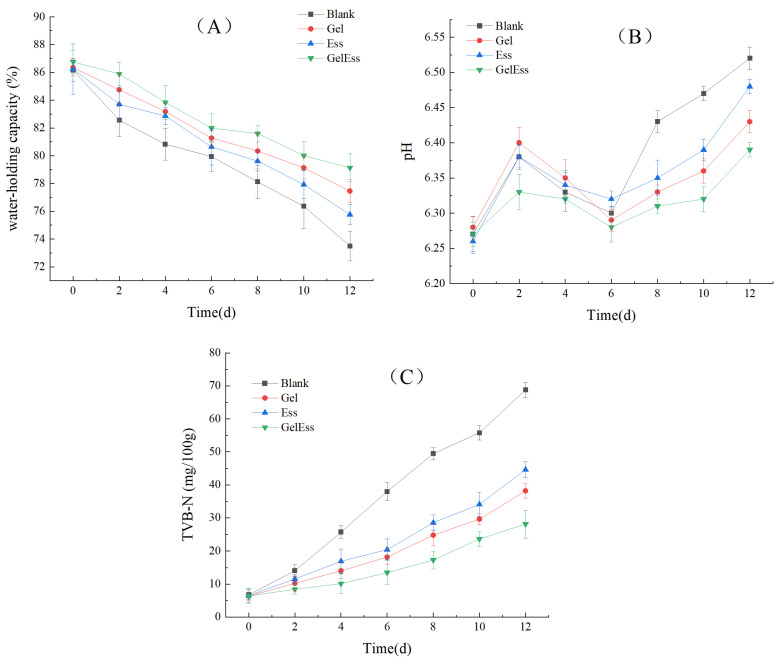
Effects of different coating methods on the water-holding capacity (**A**), pH values (**B**), and TVB-N values (**C**) of refrigerated squid muscle.

Water-holding capacity (WHC) can explain the degree of degeneration and destruction of myofibrillar protein in muscle tissue, and it is one of the important indices to evaluate the freshness of squid muscle tissue [9]. As shown in Figure 1A, the water-holding capacity of squid samples in all groups decreased significantly (*p* < 0.05). It indicated that the squid quality declined with the extension of refrigeration time. This observation aligns with previous findings by Zhu et al. [25] regarding WHC reduction in chilled squid tissues. On day 0, the water retention levels of all groups were close, and there was no significant difference among them (*p* > 0.05). It indicated that different coating methods had little effect on water retention on day 0. However, with the extension of refrigeration time, the water retention rate in the blank group was the lowest, which decreased by 12.66% on day 12 of cold storage. On the other hand, the water retention in the Gel and Ess groups slowly reduced by 8.90% and 10.47% (*p* < 0.05), respectively, during 0–12 days. The decline rate in the Gel + Ess group slowly reduced from 86.75% on day 0 to 79.13% on day 12, and the retention power decreased by 7.62% (*p* < 0.05). This might be because the Gel + Ess effectively blocked the infiltration of oxygen and inhibited the hydrolysis of lipids and protein of squid muscle during refrigeration. Furthermore, the film layer also blocked the outflow of moisture. In summary, Gel, Ess, and Gel + Ess treatments had preservation and water-hold functions, which helped to slow down the hydraulic loss, among which Gel + Ess had the best effect of delaying the decrease in water.

#### 3.1.2. pH

As shown in Figure 1B, the pH values in each group showed an increasing trend, then decreased, and finally increased. On day 0, the initial pH value in all groups was close to 6.27. There was no significant difference between them (*p* > 0.05), indicating that various coating methods had little influence on pH. The pH value increased at 0–2 days, possibly due to the destruction of squid tissue structure and the increase in alkaline substances in the early refrigeration stage. During 2–6 days, as the refrigeration time continued to increase, muscle glycogen in squid tissues decomposed and oxidized. It produced acidic substances, resulting in a decrease in the pH value. On the 6th day, the pH value dropped to the lowest (6.32 to 6.35). After 6 days, most microorganisms grew and multiplied in the squid muscle, which deteriorated the squid muscle and accumulated a large amount of alkaline substances, increasing the pH value again. The rise in pH might be caused by the production of ammonia and bioamines by microbial processes and the accumulation of basic amino acids (such as histidine) from protein hydrolysis [22]. These results were consistent with the report for refrigerated squid by Zhang et al. [26]. During the refrigeration period, the pH value of the Gel + Ess group was lower than that of the other three groups (*p* < 0.05) except for day 0, and the change range was relatively small. After 6 days of cold storage, the pH value of the other three groups increased rapidly except for the Gel + Ess group. It might be due to the microorganisms on the surface, resulting in continuous deterioration of squid muscle. However, the Gel + Ess coating might effectively inhibit the growth of microorganisms. It reduced the accumulation of alkaline nitrogen-containing compounds such as ammonia and bioamines, thus delaying the increase in pH values. Therefore, the Gel + Ess coating might inhibit protein degradation on the 2nd to 6th day of refrigeration and microbial activity on the 6th to 15th day of refrigeration, thus inhibiting the pH value of squid meat during cold storage.

#### 3.1.3. TVB-N

TVB-N content is an important indicator to characterize the degree of protein degradation and evaluate the freshness of squid muscle products. Figure 1C shows the effects of different coating methods on the TVB-N value in the prepared squid for refrigeration. The TVB-N values in all groups showed a significant increasing trend with the extension of the refrigeration time (*p* < 0.05). On day 0, the starting TVB-N value of the blank group was 6.74 mg/100 g. The TVB-N values in the Gel group, Ess group, and Gel + Ess group were 6.34, 6.46, and 6.34 mg/100 g, which were lower than those of the blank group. All of them did not reach the standard of superior grade (TVB-N < 15 mg/100 g). The results were consistent with the previous report for half-shell scallop by Zhan et al. [27]. Among them, the TVB-N value of the blank group increased the fastest with the increase in refrigeration time, reaching 37.96 mg/100 g on the 6th day. It exceeded the limit value of cephalopods TVB-N of 30 mg/100 g stipulated in the Hygienic Standard for Fresh and Frozen Animal Aquatic Products (GB 2733-2005). The value of TVB-N in the Gel group reached 29.60 mg/100 g on day 10, 28.55 mg/100 g on day 8, and 28.10 mg/100 g on day 12 in the Ess group, indicating that the value of TVB-N in both coating groups could be delayed. Among them, the most effective was the Gel + Ess group, which did not exceed the limit value after 12 days of cold storage. The increase in the TVB-N value in the refrigeration process was related to the activities of microorganisms and endogenous enzymes. The TVB-N content accumulated continuously with the growth of microorganisms. Gel + Ess coating could effectively inhibit the growth of microorganisms and reduce the production rate of volatile nitrogen during refrigeration.

#### 3.1.4. Color Change

**Figure 2:**
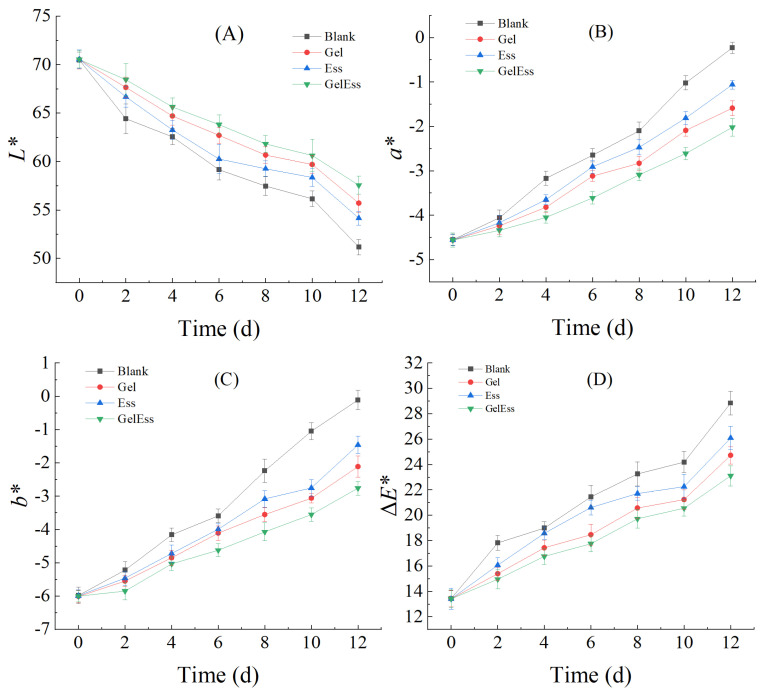
Effects of different coating methods on the color of refrigerated squid muscle. Note: (**A**) L* (0, black; 100, white); (**B**) a* (−a*: greenness, +a*: redness); (**C**) b* (−b*, blueness; +b*, yellowness); (**D**) ΔE*, overall color change.

Appearance and pleasantness play a vital role in product acceptability and preference, especially for aquatic products [9]. On day 0 of cold storage, the values of L*, a*, b*, and ΔE* among all groups were similar without significant differences (*p* > 0.05). However, there were significant differences in the values of L*, a*, b*, and ΔE* among all groups on the 2nd day of refrigeration time (*p* < 0.05). As shown in Figure 2, with the extension of refrigeration time, the L* value showed a downward trend, and the brightness of squid samples gradually decreased and lost the original luster. This might be due to the loss of water during refrigeration, which led to an increase in refractive index and a decrease in light transmission. Thus, it reduced the brightness of squid muscle surfaces and darkened their color. The L* value of the blank group decreased the fastest, followed by the Ess group and the Gel group, and the Gel + Ess group had the gentlest decline rate. It indicated that Gel + Ess significantly inhibited the decrease in the L* value of refrigerated squid muscle. With the extension of refrigeration time, the a* and b* values showed a gradual increasing trend. It indicated that the color of squid became yellow with the increase in refrigeration time. It might be due to the Browning reaction under the action of endogenous enzymes [28]. With the extension of refrigeration time, the ΔE* value showed a gradually increasing trend. The changes in the L*, a*, b*, and ΔE* values in the Gel + Ess group were relatively gentle among all groups, indicating that the preservation method of Gel + Ess coating was more helpful in maintaining the gloss of the meat quality of refrigerated squid muscle.

#### 3.1.5. Texture Profile

**Figure 3:**
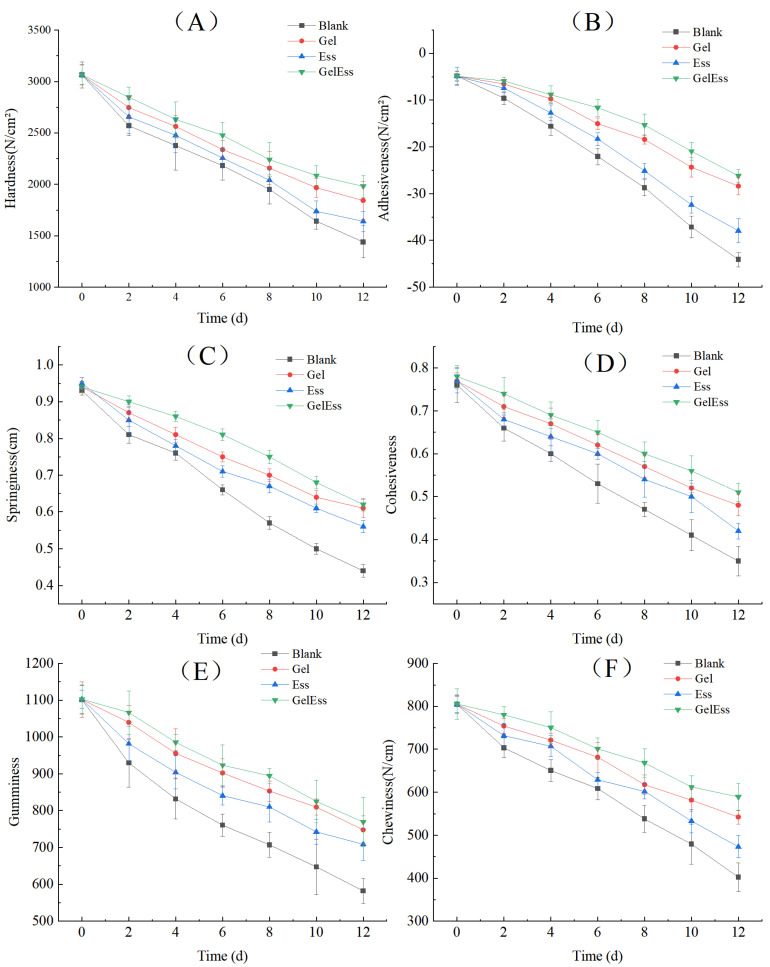
Effects of different coating methods on the texture profile of refrigerated squid muscle. Note: (**A**) Hardness (N/cm^2^); (**B**) Adhesiveness (N/cm^2^); (**C**) Springiness (cm); (**D**) Cohesiveness; (**E**) Gumminess; (**F**) Chewiness (N/cm).

According to Huo et al. [29], texture properties, including elasticity and chewiness ability, varied greatly depending on the processing and storage conditions of squids. Hardness denotes the intrinsic resistance of a material to permanent shape alteration under compressive stress. Cohesiveness quantifies the structural integrity maintenance capacity during mechanical deformation. Gumminess characterizes the intercellular adhesive forces governing tissue matrix continuity. Chewiness represents the mastication energy expenditure required to achieve swallowable consistency in solid matrices. Springiness measures the degree of structural recovery post-deformation upon force removal. Recovery indicates the rapidity of dimensional restoration following compressive deformation. Figure 3 shows the effect of different coating methods on the texture structural characteristics of refrigerated squid muscle. The six index parameters of squid muscle, including hardness, adhesiveness, springiness, cohesiveness, gumminess, and chewiness, were significantly decreased (*p* < 0.05) with the extension of refrigeration time. It indicated that the texture of squid muscle changed, and the quality decreased with the extension of refrigeration time. The decrease in these parameters during refrigeration might be caused by lipid oxidation, protein oxidation and aggregation, and water migration [2]. These results were consistent with slight oxidative and phosphate curing treatments for squid [2]. On day 0, the texture index values of each group were similar, and there was no significant difference between them (*p* > 0.05). This indicated that in the early stage of refrigeration, the coating treatment had a relatively small impact on the texture of squid muscle. The textural profile of squid manifested a progressive decline in hardness correlating with extended refrigeration duration. This phenomenon is primarily attributed to the persistently elevated endogenous enzymatic activity in squid muscle, which initiates a cascade of proteolytic events leading to structural protein degradation and concurrent lipid peroxidation processes. The blank group showed the most significant downward trend (*p* < 0.05), while the Gel + Ess group showed a slower downward trend. In summary, the Gel + Ess coating treatment has a better preservation effect on refrigerated squid muscle with less muscle quality damage, and it is more conducive to maintaining squid texture stability.

This study conducted a comprehensive evaluation of three coating treatments (Gel, Ess, and their composite Gel + Ess) on the textural attributes of refrigerated squid musculature. It found that the Gel + Ess coating had the best preservation effect, and it improved the texture quality of refrigerated squid muscle.

### 3.2. Changes in the Volatile Flavor of Refrigerated Squid Muscle

Squid muscle samples with refrigeration periods of 0, 6, and 12 days were selected to determine the changes in volatile compounds. Table S1 shows the effects of different coating methods on the volatile components of squid muscle refrigerated for 0, 6, and 12 days. Table 1 shows the ROAV of squid muscle during refrigeration in different treatment groups. A total of 113 volatile flavor compounds and 21 key flavors (ROAV > 1) were tested in the refrigerated squid muscle, of which 44, 71, and 69 volatile flavor compounds were identified at 0, 6, and 12 days, respectively. Alcohols, aldehydes, ketones, hydrocarbons, phenols, ethers, and other substances were present on day 0, day 6, and day 12, with little difference in the types of compounds (*p* > 0.05). However, there were significant differences in the relative content of each type of compound (*p* < 0.05). Multiple esters and pyridines were identified on day 0, but these two types of substances were not identified on day 6 and day 12 of cold storage. The types with relatively high content on 0, 6, and 12 days were alcohols, aldehydes, and hydrocarbons.

**Figure 4:**
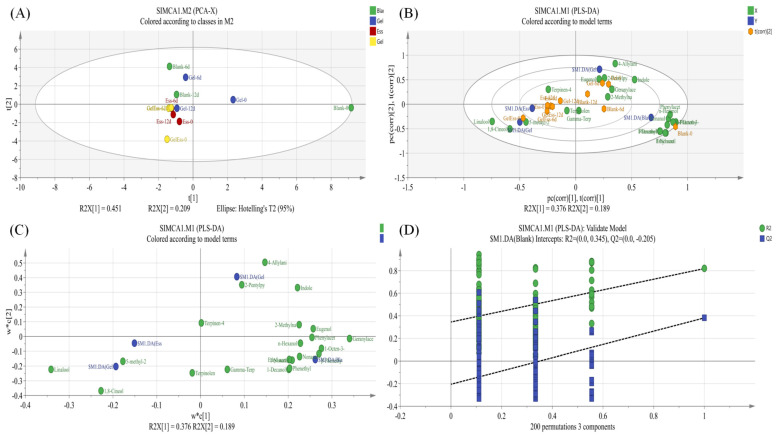
PAC analysis diagram (**A**), PLS-DA analysis (**B**,**C**), and validate model (**D**) in volatile flavor of refrigerated squid muscle pretreated with different coating methods.

As shown in Figure 4, the substances with the highest relative content in each group during a refrigeration time of 0, 6, and 12 days were alcohols. Alcohols are mainly produced by protein breakdown, amino acid metabolism, and linoleic acid degradation [30,31]. Generally, the alcohol threshold is high, but the relative content is also high; therefore, alcohols make a significant contribution to the flavor of squid muscle. On day 0, day 6, and day 12, the relative content of alcohol substances in the blank group was significantly lower than that in each coated group, especially on day 0 when the relative content of alcohol substances in the blank group reached 26.13%. This might be due to the serious damage caused to muscle tissue during the slaughter and cutting process, which reduced the activity of various endogenous enzymes and produced fewer alcohol substances. A total of 33 alcohol substances were identified, mainly including eucalyptol, alpha terpineol, and beta phenylethanol. Among them, linalool and 4-terpineol had relatively high contents, linalool had a strong and sweet green wood aroma, while 4-terpineol had a special and pleasant aroma that gave squid muscle a unique flavor. The relative content of linalool and 4-terpenol in the blank group was lower than that in the coated group, indicating that the coated group had a significant impact on the flavor compounds of refrigerated squid muscle. Interestingly, 2-methyl-3-(1-methylethenyl)-,(1R,2S,3S)-rel-cyclohexanol were only found in Gel + Ess samples after 12 days. It might be derived from the gradual degradation of terpenoids in tea tree essential oil under the action of microorganisms.

Aldehydes usually originate from the oxidation of unsaturated fatty acids, and have a relatively low threshold, contributing to the flavor of the product significantly [32]. In this experiment, a total of 17 aldehydes were detected, mainly including benzaldehyde, nonanal, phenylacetaldehyde, and 2-isopropyl-5-methyl-2-hexenal. Among them, nonanal and benzaldehyde had relatively high contents in each group. Benzyl alcohol had a bitter almond flavor, while nonanal mainly had a strong oil aroma with a hint of rose aroma, contributing greatly to the refrigerated squid muscle flavor. The relative content of aldehydes in the blank group was higher than that in the coated group on day 0, day 6, and day 12. Especially on day 0, the aldehyde content in the blank group reached 30.52%, while the content of Gel, Ess, and Gel + Ess group were lower than 1.5%. The aldehyde content in the Gel + Ess group was the lowest, followed by the Gel group and the Ess group. There was a significant difference in the relative content between the groups (*p* < 0.05). It can be inferred that the coating treatment group may inhibit the oxidation of fatty acids by microorganisms. Coating treatments reduce the production of harmful substances such as aldehydes. Gel + Ess coating has the most obvious antibacterial effect, and it can effectively inhibit microbial activity.

The ketones in squid mainly come from the thermal oxidation or degradation of unsaturated fatty acids [33], and their impact on flavor is lower than alcohols and aldehydes. A total of 11 ketone substances were detected in this experiment, among which 5-dodecyldihydro-2-(3H)-furanone, acetophenone, and 2-nonanone had relatively high relative contents. Acetophenone had a sweet and spicy taste, while 5-dodecyldihydro-2-(3H)-furanone had a strong caramel flavor. Ketones generally have a high threshold and do not contribute significantly to the flavor of refrigerated squid. The relative content of ketones in the blank group was higher than that in the coated group on day 0, day 6, and day 12. On day 0, the ketone content in the blank group was 6.59%, while that in the Gel group was 0.66%, the Ess-coated group was 0.46%, and the Gel + Ess group was 0.63%. The relative content of ketones in the coated group was much lower than that in the blank group. Overall, it indicates that coating treatment can inhibit the thermal oxidation or degradation of unsaturated fatty acids and reduce the production of ketones.

The esters in squid are produced by the interaction between alcohols and free fatty acids produced by lipid oxidation [31]. Esters have a low threshold and contribute significantly to flavor. In the present study, a total of 14 esters were identified, among which γ-nonanolactone, ethyl acetate, and methyl salicylate had relatively high relative contents. The total content of the ester substances in the blank group was higher than the coated group, and the highest relative content reached 8.23% on day 0. A total of 14 ester substances were detected on day 0, and 5 were detected on day 12. However, ester substances were not detected in the squid samples at 6 days.

The acidic substances in squid are mainly produced by fat oxidation or fat hydrolysis [34]. In this experiment, two types of acids were identified, namely acetic acid and L-2-piperidic acid. However, acid substances generally have a high threshold and contribute little to flavor. At the same time, hydrocarbons, pyridines, phenols, ethers, and some other substances were also detected. Hydrocarbons mainly come from the lipid oxidation of alkyl radicals and the decomposition of carotenoids [35]. However, the relative content of the hydrocarbon substances was relatively low, and the threshold was generally high, making a small contribution to the flavor of refrigerated squid.

In summary, Gel, Ess, and Gel + Ess coating treatments reduced the production of harmful flavor substances such as ketones and aldehydes, effectively maintaining the flavor of refrigerated squid muscle. The Gel + Ess coating holds the best effect among them, and it inhibits the volatile flavor deterioration of refrigerated squid muscle.

### 3.3. Changes in the Microorganisms of Refrigerated Squid Muscle

**Figure 5:**
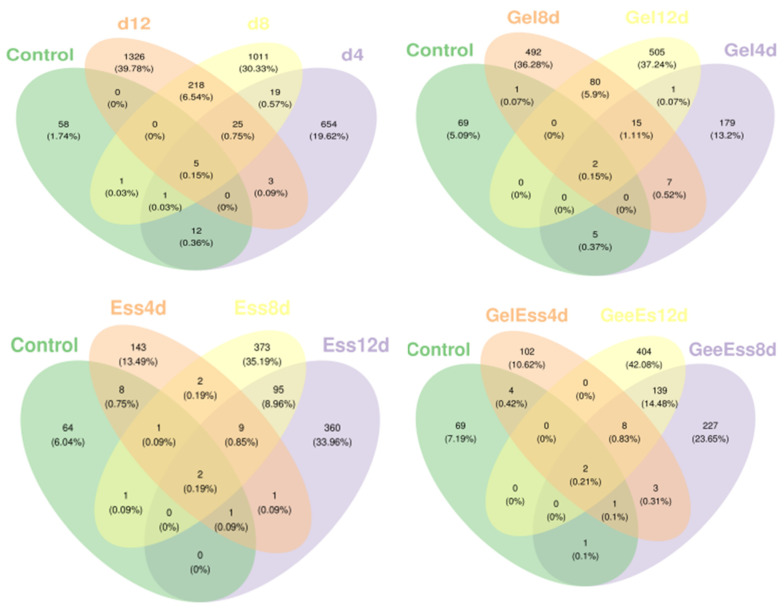
Venn diagram analysis of OTUs in refrigerated squid muscle pretreated with different coating methods.

The spoilage of refrigerated squid muscle results from bacterial metabolism, producing unnecessary metabolites. Operational taxonomic units (OTUs) sequence similarity was based on 0.97 species with a classification confidence of 0.70 from the classification database of silva138/16s_bacteria [36]. As shown in Figure 5 and Tables S2 and S3, the number of OTUs in squid samples from different groups varied over time. The total number of samples in the blank group during refrigeration was 3526. The numbers of OTUs on day 0, day 4, day 8, and day 12 were 72, 669, 1270, and 1577, respectively, accounting for approximately 1.74%, 21.57%, 38.40%, and 47.31% of the total OTUs. The common number of OTUs at different periods was 5, accounting for approximately 0.15% of the total OTUs. The total number of OTUs in the Gel group was 1501, of which the numbers of OTUs on day 0, day 4, day 8, and day 12 were 77, 209, 608, and 607, respectively, accounting for approximately 5.68%, 15.42%, 44.54%, and 44.03% of the total OTUs. The common number of OTUs in the Gel group at different periods was 2, accounting for approximately 0.15% of the total OTUs. The total number of OTUs in the Ess group was 1183. The numbers of OTUs on day 0, day 4, day 8, and day 12 were 65, 167, 483, and 468, respectively, accounting for approximately 6.45%, 19.46%, 45.56%, and 10.18% of the total OTUs. The total number of OTUs in the Ess group at different periods was 2, accounting for approximately 0.19% of the total OTUs. The total number of samples in the Gel + Ess group was 1231. The numbers of OTUs on day 0, day 4, day 8, and day 12 were 77, 120, 653, and 381, respectively, accounting for approximately 7.84%, 12.39%, 57.59%, and 39.49% of the total OTUs. The common number of OTUs in the Gel + Ess group at different periods was 2, accounting for approximately 0.21% of the total OTUs. This indicates that the number of OTUs in the blank group of squid samples increased over time with the highest total number of microbial OTUs. The number of OTUs in the other three groups also increased over time, with the Gel + Ess group showing the least changes in OTUs compared with the Gel and Ess groups.

**Figure 6:**
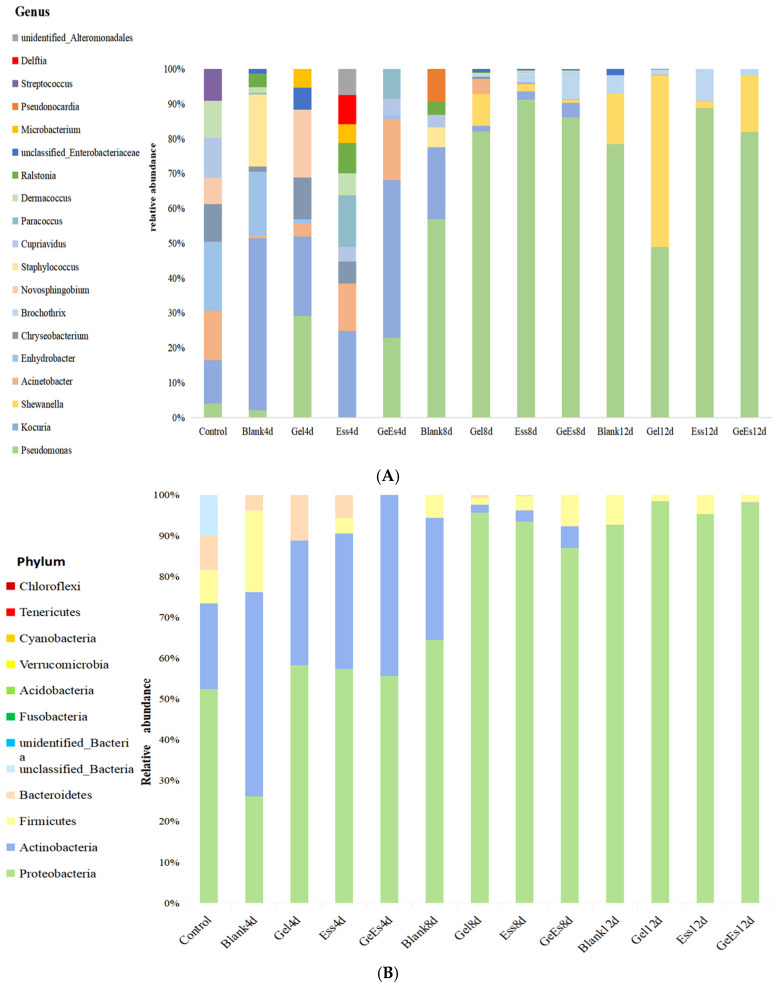
Diagram of microbial community structure in refrigerated squid muscle pretreated with different coating methods. Note: (**A**) differences of microbiomes’ genus; (**B**) differences of microbiomes’ phylum.

The unique bacterial genera in squid muscle across different groups and storage periods were analyzed (Figure 6). Notably, aerobic and anaerobic genera exhibited distinct temporal dynamics. On day 0, the dominant genera included *Staphylococcus* (facultative anaerobe), *putative Coprococcus* (strict anaerobe), *Sphingomonas* (strict aerobe), and *Streptococcus* (facultative anaerobe). On day 4, high abundances of *Streptococcus* (facultative anaerobe), *Bacteroides* (strict anaerobe), *Sphingomonas* (aerobe), and *Staphylococcus* (facultative anaerobe) were found in the blank group; *Erwinia* (facultative anaerobe), *Sphingomonas* (aerobe), *Bacteroides* (anaerobe), and *Micrococcus* (aerobe) were the dominant bacteria in the Gel group; *Microbacterium* (aerobe), *Alkalibacterium* (facultative anaerobe), *Delftia* (aerobe), *Thermophilic Bacteroides* (anaerobe), *Ralstonia* (aerobe), and *Paracoccus* (facultative anaerobe) were enriched in the Ess group; *Acinetobacter* (aerobe), *Naxibacter* (aerobe), *Paracoccus* (facultative anaerobe), and *Klebsiella* (facultative anaerobe) were predominant in the Gel + Ess group. On day 8, *Pseudoalteromonas* (facultative anaerobe) and *Bacteroides* (anaerobe) were increased in the Blank group; Clostridium (strict anaerobe), Flavobacterium (aerobe), Elizabethkingia (aerobe), and Trichomonas (anaerobe) were emergent in the Gel group. *Clostridium* (anaerobe), *Pseudomonas* (aerobe), *Lactococcus* (facultative anaerobe), and *Carnobacterium* (facultative anaerobe) were dominant in the Ess group; *Gordonia* (aerobe), *Rhodococcus* (aerobe), and *Fusobacterium* (anaerobe) were present in the Gel + Ess group. On day 12, *Ranunculus* (aerobe), *Gluconobacter* (aerobe), and *Serratia* (facultative anaerobe) were enriched in the Blank group; there was a high content of *Shewanella* (facultative anaerobe) and *Aeromonas* (facultative anaerobe) in the Gel group; *Yersinia* (facultative anaerobe) and *Fusarium* (fungal contaminant) were predominant in the Ess group; *Rhodococcus* (aerobe), *Aeromonas* (facultative anaerobe), and *Lactobacillus* (facultative anaerobe) were dominant in the Gel + Ess group.

**Figure 7:**
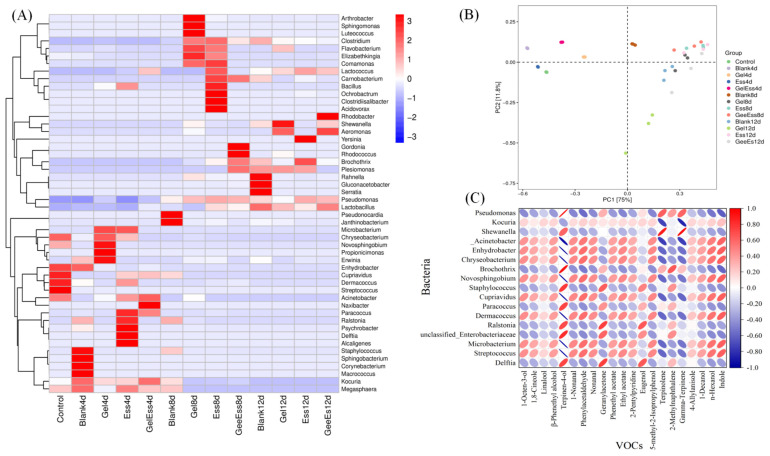
Heatmap analysis (**A**), PCA (**B**), and Pearson correlation analysis between the key flavor compounds and microorganisms (**C**) in refrigerated squid muscle pretreated with different coating methods.

The difference of microorganisms in refrigerated squid muscle with different pretreatments (Gel, Ess, Gel + Ess) was analyzed. As shown in Figure 7A,B, there was no distance between the two samples in blank groups on day 4 and day 8, and the distance was exited on day 4 and day 12; there was almost no distance between the two samples in the Gel group on day 4 and the control group, and the distance between the two samples was close on day 8 and day 12; on day 4, there was almost no close distance between the two samples in the Ess group and blank group, and there was almost no distance between the two samples in the Gel + Ess group and the blank group; on day 8, there was a close distance between the two samples in the blank group and control group, but on day 12, the distance had elongated between them. Therefore, we conclude that the blank group had a large difference in community composition, while the Ess group and Gel group had slightly higher differences in community composition than the Gel + Ess group. The Gel + Ess group holds the least difference in community composition. Altogether, the Gel + Ess coating restrains the growth of dominant spoilage bacteria such as *Corkella*, *Shewanella*, and *Bacteroides* in refrigerated squid muscle.

### 3.4. Correlation Analysis of Volatile Flavor Substances and Microorganisms in Gel + Ess Coating Squid Muscle

The Spearman’s correlation analysis (|r| > 0.60 and *p* < 0.05) between the key flavor compounds (ROAV ≥ 1, Table 1) and microorganisms (Genus, top 50, average relative abundance > 99.5%) in the cold storage process of Gel + Ess coating squid muscle were analyzed. As shown in Figure 7C, there were both high positive and negative correlations between the key flavor compounds and microorganisms. The results indicated that Terpinolene was significantly (*p* < 0.05) positively correlated with *Shewanella* and *Pseudomonas* (|r| > 0.64). The derived compounds were Terpinen-4-ol and γ-Terpinene. Terpinen-4-ol was significantly (*p* < 0.05) positively correlated with *Delftia*, *Ralstonia*, *unclassified_Enterobacteriaceae*, *Paracoccus*, *Staphylococcus*, *Brochothrix*, *Shewanella*, and *Pseudomonas* (|r| > 0.61). γ-Terpinene was significantly (*p* < 0.05) positively correlated with *Shewanella* and *Pseudomonas* (|r| > 0.61). Terpinolene is known to be a kind of monoterpene in plants widely used as an antioxidant, larvicide, and flavoring agent in various industries [37]. As shown in Figure 6, *Shewanella* and *Pseudomonas* were the dominant genus of bacteria during squid refrigeration. A similar flora was found in another study of refrigerated squid muscle [38]. The above results indicate that *Shewanella* and *Pseudomonas* might contribute to the production of these three compounds. Geranylacetone was significantly (*p* < 0.05) positively correlated with *Delftia*, *Ralstonia*, *unclassified_Enterobacteriaceae*, and *Staphylococcus* (|r| > 0.60). Geranylacetone is a volatile compound that produces a fresh grassy and fruity aroma [39]. Eugenol was significantly (*p* < 0.05) positively correlated with *Delftia* and *Ralstonia* (|r| > 0.65). Eugenol exhibited strong antimicrobial properties against various microorganisms that were used as essential oils [40]. Geranylacetone and Eugenol were terpene compounds [41], and our study suggested that *Delftia* and *Ralstonia* were the dominant terpene-producing microorganisms during squid refrigeration. 2-Methylnaphthalene was significantly (*p* < 0.05) positively correlated with *Brochothrix* (r = 0.65). 2-Methylnaphthalene was a volatile compound with a slightly pungent aroma [42]. Meanwhile, Terpinen-4-ol was significantly (*p* < 0.05) negatively correlated with *Streptococcus*, *Microbacterium*, *Dermacoccus*, *Cupriavidus*, *Novosphingobium*, *Chryseobacterium*, *Enhydrobacter*, and *Acinetobacter* (|r| > 0.95). Terpinolene was significantly (*p* < 0.05) negatively correlated with *Streptococcus*, *Microbacterium*, *Dermacoccus*, *Cupriavidus*, *Novosphingobium*, *Chryseobacterium*, *Enhydrobacter*, *Acinetobacter*, and *Kocuria* (|r| = 0.58). γ-Terpinene was significantly (*p* < 0.05) negatively correlated with *Streptococcus*, *Microbacterium*, *Dermacoccus*, *Cupriavidus*, *Novosphingobium*, *Chryseobacterium*, *Enhydrobacter*, *Acinetobacter*, and *Kocuria* (|r| = 0.54). The above results indicate that *Streptococcus*, *Microbacterium*, and *Dermacoccus* may be important microorganisms in inhibiting the production of Terpinolene compounds in the cold storage process of Gel + Ess coating squid muscle.

Interestingly, our study found that the Gel + Ess coating might inhibit flavor deterioration of refrigerated squid muscle through non-dominant spoilage bacteria (*Streptococcus*, *Microbacterium*, and *Dermacoccus*) rather than dominant spoilage bacteria (*Corkella*, *Shewanella*, *Bacteroides*). This contrasts with the findings of Tan et al. [43] (2023), who demonstrated that non-specific spoilage bacteria accelerate sturgeon muscle deterioration during late-stage refrigeration through extracellular peptidase-, lipase-, and esterase-mediated enhancement of lipid and amino acid metabolism. However, the current study has limitations that warrant consideration: (1) the precise inhibitory mechanisms of non-dominant bacteria on flavor-related metabolites remain uncharacterized; (2) functional validation of the observed microbial shifts was not performed; and (3) potential interactions between coating components and microbial communities were not systematically analyzed. Consequently, while these findings suggest a novel preservation strategy, the specific pathways by which non-dominant spoilage bacteria inhibit flavor deterioration in Gel + Ess squid muscle during refrigeration require further mechanistic investigation.

## 4. Conclusions

The impact of Gel, Ess, and Gel + Ess coating on the quality, flavor deterioration, and microbial diversity of refrigerated squid muscle was investigated. The Gel + Ess coating significantly mitigated the decline in water retention capacity, texture parameters, and sensory evaluation scores, while also reducing the increase in TVB-N values in refrigerated squid muscle. A total of 113 volatile flavor compounds were detected in refrigerated squid muscle, characterized by a high concentration of alcohols and a low concentration of aldehydes. Metagenomic analysis revealed that *Bacteroidetes*, a dominant microbial phylum, played a primary role in the pre-formed spoilage of squid. The Gel + Ess coating effectively suppressed the proliferation of *Bacteroidetes* in the refrigerated squid muscle. At the genus level, *Kochia* and *Shewanella* emerged as the predominant bacterial genera responsible for the deterioration of squid muscle. The Gel + Ess coating significantly inhibited the growth of these dominant bacterial communities. These findings demonstrate that the Gel + Ess coating is a viable and effective green preservative for refrigerated squid muscle. To advance these findings, future research should investigate the molecular interactions between coating components and bacterial membrane proteins through proteomic approaches.

## Figures and Tables

**Table 1:** The relative odor activity values (ROAV) of squid muscle during refrigeration in different treatment groups.

Name.	ROAV											
	0 Days				6 Days				12 Days			
	Blank	Gel	Ess	Gel + Ess	Blank	Gel	Ess	Gel + Ess	Blank	Gel	Ess	Gel + Ess
1-Decanol	5.06	n.d.	n.d.	n.d.	n.d.	n.d.	n.d.	n.d.	n.d.	n.d.	n.d.	n.d.
n-Hexanol	4.09	0.01	n.d.	0.00	0.02	0.25	n.d.	n.d.	n.d.	0.00	n.d.	n.d.
1-Octen-3-ol	52.29	0.09	n.d.	n.d.	1.03	71.75	n.d.	n.d.	n.d.	0.12	n.d.	n.d.
1,8-Cineole	n.d.	n.d.	25.26	21.60	100.00	n.d.	100.00	100.00	12.37	5.09	9.23	15.76
Linalool	53.11	11.38	100.00	100.00	n.d.	n.d.	n.d.	n.d.	100.00	100.00	100.00	100.00
β-Phenethylalcohol	29.65	0.03	0.01	0.00	0.19	38.87	0.03	0.01	0.28	0.05	0.01	0.00
Terpinen-4-ol	n.d.	n.d.	n.d.	n.d.	0.02	1.75	0.05	0.02	0.02	0.02	0.00	0.01
1-Nonanal	100.00	n.d.	n.d.	n.d.	0.27	n.d.	n.d.	n.d.	n.d.	n.d.	n.d.	n.d.
Phenylacetaldehyde	5.72	0.03	n.d.	n.d.	0.13	n.d.	n.d.	n.d.	n.d.	n.d.	n.d.	n.d.
Nonanal	91.30	0.13	n.d.	n.d.	n.d.	37.47	n.d.	n.d.	0.21	n.d.	n.d.	n.d.
Geranylacetone	n.d.	0.01	n.d.	n.d.	0.43	9.97	n.d.	n.d.	0.27	0.03	n.d.	n.d.
Phenethylacetate	24.33	0.00	n.d.	n.d.	n.d.	n.d.	n.d.	n.d.	n.d.	n.d.	n.d.	n.d.
Ethylacetate	2.79	n.d.	n.d.	n.d.	n.d.	n.d.	n.d.	n.d.	n.d.	n.d.	n.d.	n.d.
2-Pentylpyridine	n.d.	99.97	n.d.	n.d.	n.d.	n.d.	n.d.	n.d.	n.d.	n.d.	n.d.	n.d.
Eugenol	n.d.	n.d.	0.02	0.02	0.33	32.01	n.d.	n.d.	0.55	n.d.	n.d.	n.d.
5-methyl-2-Isopropylphenol	n.d.	n.d.	1.34	1.85	n.d.	n.d.	n.d.	n.d.	n.d.	n.d.	n.d.	n.d.
Terpinolene	n.d.	n.d.	n.d.	n.d.	0.18	3.34	0.16	0.13	n.d.	0.00	0.01	0.03
2-Methylnaphthalene	n.d.	n.d.	n.d.	n.d.	0.86	34.27	n.d.	n.d.	n.d.	0.05	n.d.	n.d.
Gamma-Terpinene	n.d.	n.d.	n.d.	n.d.	0.13	1.61	0.07	0.06	n.d.	0.00	0.00	0.01
4-Allylanisole	n.d.	0.08	n.d.	n.d.	n.d.	99.99	n.d.	n.d.	0.31	n.d.	n.d.	n.d.
Indole	0.27	0.00	0.00	0.00	0.05	7.38	0.03	0.00	0.01	0.00	0.00	0.00

Note: n.d. indicates that the compound was not detected.

## Data Availability

The original contributions presented in the study are included in the article/Supplementary Material, further inquiries can be directed to the corresponding author.

## Supplementary Materials

The following supporting information can be downloaded at: https://www.mdpi.com/article/10.3390/foods14071160/s1, Table S1: The volatile substances in squid muscle pretreated with different coating refrigerated for 0 days, 6 days, and 12 days; Table S2: Alpha diversity statistics in refrigerated squid muscle pretreated with different coating methods; Table S3: OTU statistics of refrigerated squid muscle pretreated with different coating methods.
