# Supplementary material for: Gelatin–Tea Tree Essential Oil Coating Improves the Quality, Flavor, and Micromolecular Metabolites of Squid (Illex argentinus) Muscle During Cold Storage
Source: Foods. 2025 Mar 27;14(7):1160. doi: 10.3390/foods14071160 (PMC11988927; doi:10.3390/foods14071160)
Supplement: Supplementary file 1 [file foods-14-01160-s001.zip › foods-3502332-supplementary.pdf]

Table S1. The volatile substances in squid muscle pretreated with different coating refrigerated for 0 days, 6 days, and 12 days.

Name	CAS NO.	Threshold	Relative concentration/%											
			0 days				6 days				12 days			
			Blank	Gel	Ess	Gel+Ess	Blank	Gel	Ess	Gel+Ess	Blank	Gel	Ess	Gel+Ess
**Alcohols**														
3-Methyl-1-butanol	123-51-3	0.25	n.d.	n.d.	n.d.	n.d.	n.d.	n.d.	n.d.	n.d.	0.3583	0.0687	n.d.	n.d.
1-Decanol	112-30-1	0.023	0.9068	n.d.	n.d.	n.d.	n.d.	n.d.	n.d.	n.d.	n.d.	n.d.	n.d.	n.d.
1-Dodecanol	112-53-8	0.066	n.d.	n.d.	n.d.	0.0054	n.d.	n.d.	n.d.	n.d.	n.d.	n.d.	n.d.	n.d.
n-Hexanol	111-27-3	0.2	6.3654	3.1940	n.d.	0.0671	0.3554	0.0532	n.d.	n.d.	n.d.	n.d.	n.d.	n.d.
1-Octen-3-ol	3391-86-4	0.002	0.8146	0.1918	n.d.	n.d.	0.1731	0.1545	n.d.	n.d.	n.d.	0.0328	n.d.	n.d.
1-Pentanol	71-41-0	880	0.2493	0.3431	n.d.	n.d.	n.d.	n.d.	n.d.	n.d.	n.d.	n.d.	n.d.	n.d.
2-Furanmethanol	98-00-0	1	0.1092	0.0478	n.d.	n.d.	n.d.	n.d.	n.d.	n.d.	n.d.	n.d.	n.d.	n.d.
Benzyl alcohol	100-51-6	5.5	0.6132	n.d.	n.d.	n.d.	n.d.	n.d.	0.0176	n.d.	n.d.	n.d.	0.0307	n.d.
1,8-Cineole	470-82-6	0.00026	n.d.	n.d.	1.9187	4.7020	2.1921	n.d.	1.0790	1.9543	0.3564	0.1854	1.0327	0.9252
Linalool	78-70-6	0.00022	0.0910	2.7905	6.4272	18.4183	n.d.	n.d.	n.d.	n.d.	2.4380	3.0856	9.4704	4.9666
Alpha-Terpineol	10482-56-1	0.3	n.d.	0.8470	n.d.	9.0972	1.2753	1.0725	n.d.	n.d.	1.1628	1.2121	n.d.	n.d.
Phenethyl alcohol	60-12-8	0.045	10.3946	1.7487	0.0698	0.0325	0.7255	1.8830	0.0486	0.0216	1.4107	0.2997	0.1646	0.0156

Cis-alpha,alpha,5-trimethyl-5-vinyltetrahydrofuran-2-methanol	5989-33-3	n.d.	n.d.	n.d.	n.d.	n.d.	n.d.	n.d.	n.d.	0.1904	n.d.	n.d.	0.2229	n.d.
3-(Hydroxymethyl)nonan-2-one	67801-33-6	n.d.	n.d.	n.d.	n.d.	n.d.	n.d.	n.d.	0.0208	n.d.	n.d.	n.d.	0.0388	n.d.
(2Z)-2-Octene-1-ol	26001-58-1	n.d.	n.d.	n.d.	n.d.	n.d.	0.5419	n.d.	n.d.	n.d.	n.d.	n.d.	n.d.	n.d.
4-Thujanol	546-79-2	n.d.	n.d.	n.d.	n.d.	n.d.	0.5367	n.d.	1.4476	1.1309	n.d.	0.4203	2.4966	0.8177
2-(4-Methylphenyl)propan-2-ol	1197-01-9	n.d.	n.d.	n.d.	n.d.	n.d.	n.d.	n.d.	n.d.	0.1283	n.d.	n.d.	n.d.	n.d.
L(-)-Borneol	464-45-9	0.00018	n.d.	n.d.	n.d.	n.d.	n.d.	n.d.	0.2133	n.d.	n.d.	n.d.	n.d.	n.d.
Beta-terpineol	138-87-4	n.d.	n.d.	n.d.	n.d.	n.d.	n.d.	n.d.	0.0545	0.0910	n.d.	n.d.	0.1418	n.d.
Borneol	507-70-0	n.d.	n.d.	n.d.	n.d.	n.d.	n.d.	n.d.	n.d.	0.6389	n.d.	n.d.	0.7662	0.4620
Fenchol	1632-73-1	n.d.	n.d.	n.d.	n.d.	n.d.	0.0955	n.d.	0.2845	0.7076	n.d.	0.0347	0.6139	0.5117
2-(3-Methylphenyl)propan-2-ol	5208-37-7	n.d.	n.d.	n.d.	n.d.	n.d.	n.d.	n.d.	0.1027	n.d.	n.d.	n.d.	n.d.	n.d.
Terpinen-4-ol	562-74-3	n.d.	n.d.	n.d.	n.d.	n.d.	62.0338	75.4083	78.3366	67.0193	82.5883	90.6643	73.4309	79.7410
Terpineol	8000-41-7	n.d.	n.d.	n.d.	n.d.	n.d.	n.d.	n.d.	3.9852	4.8261	n.d.	n.d.	6.7705	3.4897
(E)-Verbenol	1820-09-3	n.d.	n.d.	n.d.	n.d.	n.d.	n.d.	n.d.	0.0812	0.2399	n.d.	n.d.	0.1561	0.1735

(E)-6-(isopropyl)-3-methylcyclohex-2-en-1-ol	16721-39-4	n.d.	n.d.	n.d.	n.d.	n.d.	n.d.	n.d.	n.d.	n.d.	n.d.	n.d.	0.0401	n.d.
(E)-2-Tridecen-1-ol	74962-98-4	n.d.	n.d.	n.d.	n.d.	n.d.	n.d.	n.d.	n.d.	n.d.	0.0600	n.d.	n.d.	n.d.
1-octen-3-ol	64275-73-6	n.d.	n.d.	n.d.	n.d.	n.d.	n.d.	n.d.	n.d.	n.d.	0.4802	0.2011	0.0229	n.d.
2-methyl-3-(1-methylethenyl)-,(1R,2S,3S)-rel-Cyclohexanol	54244-81-4	n.d.	n.d.	n.d.	n.d.	n.d.	n.d.	n.d.	n.d.	n.d.	n.d.	n.d.	n.d.	0.0272
**Aldehydes**														
1-Nonanal	143-08-8	0.002	1.5579	n.d.	n.d.	n.d.	0.0455	n.d.	n.d.	n.d.	n.d.	n.d.	n.d.	n.d.
Phenylacetaldehyde	122-78-1	0.009	0.4008	n.d.	n.d.	n.d.	0.0957	n.d.	n.d.	n.d.	n.d.	n.d.	n.d.	n.d.
Decanal	112-31-2	0.005	n.d.	0.0408	n.d.	n.d.	n.d.	n.d.	n.d.	n.d.	n.d.	n.d.	n.d.	n.d.
Nonanal	124-19-6	0.0035	2.4892	0.4886	n.d.	n.d.	n.d.	0.1412	n.d.	n.d.	0.0809	n.d.	n.d.	n.d.
5-Methyl-2-isopropyl-2-hexenal	35158-25-9	n.d.	n.d.	n.d.	n.d.	n.d.	n.d.	n.d.	0.0169	0.0339	n.d.	n.d.	n.d.	0.0245
Benzaldehyde	100-52-7	0.3	n.d.	n.d.	n.d.	n.d.	n.d.	n.d.	0.0910	n.d.	0.9407	n.d.	n.d.	n.d.
Dodecyl aldehyde	112-54-9	17	n.d.	n.d.	n.d.	n.d.	0.0825	n.d.	n.d.	n.d.	n.d.	n.d.	n.d.	n.d.

Ketones														
2-Decanone	693-54-9	156	n.d.	n.d.	n.d.	0.0363	n.d.	n.d.	n.d.	n.d.	n.d.	n.d.	n.d.	n.d.
2-Piperidone	675-20-7	300	n.d.	n.d.	0.0057	0.0023	0.0393	0.1635	0.0030	n.d.	n.d.	n.d.	n.d.	n.d.
Geranylacetone	3796-70-1	0.01	n.d.	0.0745	n.d.	n.d.	0.3602	0.1074	n.d.	n.d.	0.2957	0.0472	n.d.	n.d.
6-Methyl-5-hepten-2-one	110-93-0	0.1	n.d.	n.d.	0.0159	n.d.	n.d.	n.d.	n.d.	n.d.	n.d.	n.d.	n.d.	n.d.
2-Nonanone	821-55-6	0.2	n.d.	n.d.	n.d.	n.d.	n.d.	n.d.	n.d.	0.0260	0.4877	0.2033	n.d.	0.0188
4-Methyl-2(5H)-furanone	6124-79-4	n.d.	n.d.	n.d.	n.d.	n.d.	n.d.	0.4451	n.d.	n.d.	n.d.	n.d.	n.d.	n.d.
Acetophenone	98-86-2	3.0	n.d.	n.d.	n.d.	n.d.	0.1600	0.2931	0.1222	0.0922	0.3083	0.0504	0.0863	0.0667
5-Isopropylbicyclo[3.1.0]Hexan-2-One	513-20-2	n.d.	n.d.	n.d.	n.d.	n.d.	n.d.	n.d.	n.d.	0.0152	n.d.	n.d.	0.0283	0.0110
2-Tridecanone	593-08-8	n.d.	n.d.	n.d.	n.d.	n.d.	n.d.	n.d.	n.d.	n.d.	n.d.	0.0469	n.d.	n.d.
Nerylacetone	3879-26-3	n.d.	n.d.	n.d.	n.d.	n.d.	n.d.	n.d.	n.d.	n.d.	n.d.	n.d.	0.0130	0.0066
Camphor	76-22-2	n.d.	n.d.	n.d.	n.d.	n.d.	n.d.	n.d.	n.d.	n.d.	0.4501	n.d.	n.d.	n.d.
**Esters**														
γ-Decalactone	706-14-9	0.064	0.4681	n.d.	n.d.	n.d.	n.d.	n.d.	n.d.	n.d.	n.d.	n.d.	n.d.	n.d.
γ-Nonanolactone	104-61-0	1	5.4387	0.1778	n.d.	n.d.	n.d.	n.d.	n.d.	n.d.	n.d.	n.d.	n.d.	n.d.
Phenethy lacetate	103-45-7	0.02	3.7904	0.0654	n.d.	n.d.	n.d.	n.d.	n.d.	n.d.	n.d.	n.d.	n.d.	n.d.
Ethy lacetate	142-92-7	0.04	0.8694	n.d.	n.d.	n.d.	n.d.	n.d.	n.d.	n.d.	n.d.	n.d.	n.d.	n.d.

Methyl salicylate	119-36-8	0.06	n.d.	n.d.	0.2994	0.3198	n.d.	n.d.	n.d.	n.d.	n.d.	0.1475	0.1220	0.1952
(Z)-2-Butenoicacid ethyl ester	6776-19-8	n.d.	n.d.	n.d.	n.d.	n.d.	n.d.	n.d.	n.d.	n.d.	n.d.	0.0531	n.d.	n.d.
Ethyl hex-2-enoate	1552-67-6	n.d.	n.d.	n.d.	n.d.	n.d.	n.d.	n.d.	n.d.	n.d.	0.5098	0.1221	n.d.	n.d.
1-Terpinen-4-ylacetate	4821-04-9	n.d.	n.d.	n.d.	n.d.	n.d.	n.d.	n.d.	n.d.	n.d.	0.0866	n.d.	n.d.	n.d.
Isoamyl isovalerate	659-70-1	0.02	n.d.	n.d.	n.d.	n.d.	n.d.	n.d.	n.d.	n.d.	n.d.	0.0180	n.d.	0.1606
**Acids**														
Acetic acid	64-19-7	5500	2.5338	1.8224	0.1159	n.d.	n.d.	n.d.	n.d.	n.d.	n.d.	n.d.	n.d.	n.d.
L(-)-Pipecolinic acid	3105-95-1	3230	n.d.	n.d.	n.d.	0.0103	n.d.	n.d.	n.d.	n.d.	n.d.	n.d.	n.d.	n.d.
**Hydrocarbons**														
(+)-P-menth-1-ene	18368-95-1	0.0393	n.d.	n.d.	0.0559	n.d.	n.d.	n.d.	n.d.	n.d.	n.d.	n.d.	n.d.	n.d.
Dipentene	5989-27-5	0.034	n.d.	n.d.	n.d.	0.3929	n.d.	n.d.	n.d.	n.d.	n.d.	n.d.	n.d.	n.d.
Styrene	100-42-5	0.022	n.d.	0.2165	n.d.	n.d.	0.5753	1.1941	n.d.	n.d.	1.2268	n.d.	0.0824	n.d.
Myrcene	123-35-3	0.0166	n.d.	n.d.	n.d.	0.2524	n.d.	n.d.	n.d.	n.d.	n.d.	n.d.	n.d.	n.d.
3-Methylene-6-(1-methylethyl)-Cyclohexene	555-10-2	0.036	n.d.	n.d.	n.d.	0.1127	n.d.	n.d.	n.d.	0.0704	n.d.	n.d.	0.2022	0.0344

Pyridine	110-86-1	80	0.2669	n.d.	n.d.	n.d.	n.d.	n.d.	n.d.	n.d.	n.d.	n.d.	n.d.	n.d.
2-Pentylpyridine	2294-76-0	0.0000012	n.d.	0.1337	n.d.	n.d.	n.d.	n.d.	n.d.	n.d.	n.d.	n.d.	n.d.	n.d.
Eugenol	97-53-0	0.001	n.d.	n.d.	0.0045	0.0185	0.0282	0.0345	n.d.	n.d.	0.0609	n.d.	n.d.	n.d.
P-Cresol	106-44-5	0.002	n.d.	n.d.	0.0099	0.0215	n.d.	n.d.	0.0095	0.0064	n.d.	n.d.	0.0090	0.0046
Phenol	108-95-2	5.5	0.5737	1.1991	0.2646	0.0862	1.7932	3.5994	0.3879	0.1478	2.2362	0.2471	0.4555	0.1068
5-Methyl-2-Isopropylphenol	499-75-2	0.0001	n.d.	n.d.	0.0391	0.1549	n.d.	n.d.	n.d.	n.d.	n.d.	n.d.	n.d.	n.d.
1,3,5,5-Tetramethyl-1,3-cyclohexadiene	4724-89-4	n.d.	n.d.	n.d.	n.d.	n.d.	n.d.	0.0524	n.d.	n.d.	n.d.	n.d.	n.d.	n.d.
NeoAlloociMene	7216-56-0	n.d.	n.d.	n.d.	n.d.	n.d.	n.d.	n.d.	n.d.	0.2472	n.d.	n.d.	n.d.	n.d.
2,6-Dimethyl-2,4,6-octariene	57396-75-5	n.d.	n.d.	n.d.	n.d.	n.d.	n.d.	n.d.	0.0271	n.d.	n.d.	n.d.	n.d.	n.d.
3,5,5-Trimethyl-2-hexene	26456-76-8	n.d.	n.d.	n.d.	n.d.	n.d.	n.d.	0.3375	n.d.	n.d.	n.d.	n.d.	n.d.	n.d.
3-Carene	13466-78-9	0.77	n.d.	n.d.	n.d.	n.d.	n.d.	n.d.	n.d.	0.1448	n.d.	n.d.	n.d.	0.1047
6,7,8,9-tetrahydro-5H-benzo[7]annulene	1075-16-7	n.d.	n.d.	n.d.	n.d.	n.d.	0.0287	n.d.	n.d.	n.d.	n.d.	n.d.	n.d.	n.d.

1-Isopropenyl-3-methylbenzene	1124-20-5	n.d.	n.d.	n.d.	n.d.	n.d.	n.d.	n.d.	0.7521	0.0493	0.8749	0.6257	0.7920	0.0357
M-cymene	535-77-3	n.d.	n.d.	n.d.	n.d.	n.d.	n.d.	0.6924	n.d.	n.d.	0.8032	0.0933	n.d.	n.d.
alpha,para-Dimethylstyrene	1195-32-0	n.d.	n.d.	n.d.	n.d.	n.d.	n.d.	1.1444	n.d.	1.6470	n.d.	n.d.	n.d.	1.1909
4-Ethenyl-1,2-dimethyl-benzene	27831-13-6	n.d.	n.d.	n.d.	n.d.	n.d.	0.8794	n.d.	n.d.	n.d.	n.d.	n.d.	n.d.	n.d.
Sabinene	3387-41-5	0.98	n.d.	n.d.	n.d.	n.d.	1.4695	n.d.	0.1848	0.3415	n.d.	n.d.	n.d.	0.1420
Terpinolene	586-62-9	0.2	n.d.	n.d.	n.d.	n.d.	3.0437	0.7192	1.3112	1.9379	n.d.	0.1311	0.6981	1.4012
(R)-1-Methyl-5-(1-methylvinyl)cyclohexene	1461-27-4	n.d.	n.d.	n.d.	n.d.	n.d.	n.d.	n.d.	n.d.	0.0194	n.d.	n.d.	n.d.	0.0128
1-Methylnaphthalene	90-12-0	0.02	n.d.	n.d.	n.d.	n.d.	n.d.	n.d.	n.d.	n.d.	0.2188	n.d.	0.0069	n.d.
3-Methyl-6-(1-methylethylidene)cyclohexene	91-57-6	0.003	n.d.	n.d.	n.d.	n.d.	0.2164	0.1107	n.d.	n.d.	n.d.	0.0228	n.d.	n.d.
2-Methylnaphthalene	527-84-4	n.d.	n.d.	n.d.	n.d.	n.d.	4.6321	1.2350	0.4460	0.6684	n.d.	n.d.	0.3745	0.4833

β-pinene	127-91-3	n.d.	n.d.	n.d.	n.d.	n.d.	n.d.	n.d.	0.2257	0.4876	n.d.	n.d.	0.0567	0.2566
γ-Terpinene	99-85-4	1	n.d.	n.d.	n.d.	n.d.	10.7215	1.7326	3.0813	4.5548	n.d.	0.3474	0.9362	3.2300
2,4-Di-tert-butylphenol	96-76-4	n.d.	n.d.	n.d.	n.d.	n.d.	1.2678	1.4076	0.0661	0.0470	n.d.	n.d.	n.d.	0.0340
2-Tert-butyl-6-methylphenol	2219-82-1	n.d.	n.d.	n.d.	n.d.	n.d.	0.0883	n.d.	n.d.	n.d.	n.d.	n.d.	n.d.	n.d.
(Z)-anethole	25679-28-1	n.d.	n.d.	n.d.	n.d.	n.d.	n.d.	n.d.	n.d.	n.d.	1.2651	n.d.	n.d.	n.d.
3-Methyl-4-isopropylphenol	3228-02-2	n.d.	n.d.	n.d.	n.d.	n.d.	n.d.	n.d.	n.d.	n.d.	n.d.	0.0216	n.d.	n.d.
2-Tert-Butyl-4-methylphenol	2409-55-4	0.03	n.d.	n.d.	n.d.	n.d.	n.d.	n.d.	n.d.	n.d.	n.d.	0.2421	n.d.	n.d.
4-Tert-butyl-2-methylphenol	98-27-1	0.4	n.d.	n.d.	n.d.	n.d.	n.d.	n.d.	n.d.	n.d.	0.5491	n.d.	n.d.	n.d.
Thymol	89-83-8	1.7	n.d.	n.d.	n.d.	n.d.	n.d.	n.d.	n.d.	n.d.	n.d.	n.d.	0.0062	n.d.
Ethers														
4-Allylanisole	140-67-0	0.0075	n.d.	0.6471	n.d.	n.d.	n.d.	0.8074	n.d.	n.d.	0.2573	n.d.	n.d.	n.d.
Acetamide	60-35-5	5900	n.d.	n.d.	0.0168	n.d.	n.d.	n.d.	0.0877	0.0299	n.d.	n.d.	n.d.	n.d.
Anethol	104-46-1	0.1	n.d.	n.d.	0.8513	1.7072	0.0667	n.d.	0.6857	1.5639	n.d.	0.5927	0.3447	0.9381
(-)-Camphor	464-48-2	380	n.d.	n.d.	3.1468	10.6467	1.0878	0.1623	2.0833	2.5916	n.d.	0.3273	n.d.	n.d.

Formamide	75-12-7	4500	n.d.	0.0837	n.d.	n.d.	n.d.	n.d.	n.d.	n.d.	n.d.	n.d.	n.d.	n.d.
Indole	120-72-9	0.5	1.0443	1.6962	0.4202	0.2108	2.2530	3.9724	0.5598	0.1684	0.3915	0.0715	0.1096	0.1218
Quinine	130-95-0	31	0.0723	n.d.	n.d.	n.d.	n.d.	n.d.	n.d.	n.d.	n.d.	n.d.	n.d.	n.d.
3,4-Diaminotoluene	496-72-0	n.d.	n.d.	n.d.	n.d.	n.d.	n.d.	0.1401	n.d.	n.d.	n.d.	n.d.	n.d.	n.d.
4-Methylimidazole	822-36-6	n.d.	n.d.	n.d.	n.d.	n.d.	n.d.	0.1532	n.d.	n.d.	n.d.	n.d.	n.d.	n.d.
Fema3661	1786-08-9	n.d.	n.d.	n.d.	n.d.	n.d.	n.d.	n.d.	0.0955	n.d.	n.d.	0.1783	n.d.	n.d.
4,7-Dimethylbenzofuran	28715-26-6	n.d.	n.d.	n.d.	n.d.	n.d.	n.d.	0.0706	0.1010	0.1832	n.d.	n.d.	n.d.	n.d.
o-Tolunitrile	529-19-1	n.d.	n.d.	n.d.	n.d.	n.d.	n.d.	n.d.	0.0101	0.0081	n.d.	n.d.	n.d.	0.0059
1-Acetyl-2-methylcyclopentene	3168-90-9	n.d.	n.d.	n.d.	n.d.	n.d.	n.d.	n.d.	0.1085	n.d.	n.d.	n.d.	0.0891	n.d.
Glafenine	3820-67-5	n.d.	n.d.	n.d.	n.d.	n.d.	0.1035	n.d.	n.d.	n.d.	n.d.	n.d.	n.d.	n.d.
Mefluidide	53780-34-0	n.d.	n.d.	n.d.	n.d.	n.d.	n.d.	n.d.	n.d.	0.0120	n.d.	n.d.	n.d.	n.d.
1-Allyl-3,4-methylidenedioxybenzene	94-59-7	0.16	n.d.	n.d.	n.d.	n.d.	0.1305	0.0164	0.2201	0.3924	0.1018	0.1806	0.1371	0.2837
Benzo[d]Isothiazole	272-16-2	n.d.	n.d.	n.d.	n.d.	n.d.	n.d.	n.d.	n.d.	n.d.	n.d.	0.0252	n.d.	n.d.
Benzoylhydrazine	613-94-5	n.d.	n.d.	n.d.	n.d.	n.d.	n.d.	n.d.	n.d.	n.d.	n.d.	n.d.	0.0403	n.d.

Phenylacetonitrile	140-29-4	1.2	n.d.	n.d.	n.d.	n.d.	n.d.	n.d.	n.d.	n.d.	n.d.	n.d.	0.0109	n.d.

Table S2. Alpha diversity statistics in refrigerated squid muscle pretreated with different coating methods.

Sample	Chao1	simpson	shannon	Pielou
Control	51.70	0.89	14.02	0.63
Blank4d	170.52	0.81	8.02	0.51
Blank8d	41.98	0.88	11.02	0.67
Blank12d	298.05	0.84	25.02	0.50
Gel4d	114.63	0.92	35.52	0.67
Gel8d	262.14	0.93	37.02	0.65
Gel12d	348.06	0.94	41.02	0.61
Ess4ds	93.70	0.91	20.02	0.62
Ess8ds	284.58	0.90	25.52	0.57
Ess12d	343.68	0.91	17.02	0.58
GeEss4d	68.55	0.84	28.52	0.57
GeEss8d	68.55	0.85	31.52	0.56
GeEss12d	222.37	0.87	22.52	0.59

Table S3. OTU statistics of refrigerated squid muscle pretreated with different coating methods.

Sample	Taxon Tags	OTU
Control	72328	250
Blank4d	72328	250
Blank8d	69514	171
Blank12d	70424	132
Gel4d	78009	90
Gel8d	85963	42
Gel12d	48670	429
Ess4ds	30679	311
Ess8ds	41026	186
Ess12d	39902	432
GeEss4d	38369	323
GeEss8d	53439	198
GeEss12d	52883	255
